# Characterization of non-cardiac arrest PulsePoint activations in public and private settings

**DOI:** 10.1186/s12873-023-00849-z

**Published:** 2023-07-27

**Authors:** Jennifer Blackwood, Mohamud R. Daya, Ben Sorenson, Brian Schaeffer, Mike Dawson, Michael Charter, James Mark Nania, Julie Charbonneau, Jeremy Robertson, Michael Mancera, Chris Carbon, Dawn B. Jorgenson, Mengqi Gao, Richard Price, Chris Rosse, Thomas Rea

**Affiliations:** 1 Seattle & King County Public Health, 401 5th Ave, Suite 1200, Seattle, WA 98104 USA; 2 Oregon Health & Sciences University, Portland, OR USA; 3 Tualatin Valley Fire & Rescue, Tigard, OR USA; 4 City of Spokane Fire Dept, Spokane, WA USA; 5 Spokane Valley Fire, Spokane Valley, WA USA; 6 Spokane County EMS Office, Spokane, WA USA; 7 Sioux Falls Fire & Rescue, Sioux Falls, SD USA; 8 University of Wisconsin-Madison, Madison, WI USA; 9 City of Madison Fire Department, Madison, WI USA; 10 Philips Medical, Bothell, WA USA; 11 PulsePoint Foundation, Pleasanton, CA USA; 12 University of Washington, Seattle, WA USA

**Keywords:** Out-of-hospital cardiac arrest, Social media, Crowdsourcing, Emergency medical services, Prehospital

## Abstract

**Background:**

Geospatial smartphone application alert systems are used in some communities to crowdsource community response for out-of-hospital cardiac arrest (OHCA). Although the clinical focus of this strategy is OHCA, dispatch identification of OHCA is imperfect so that activation may occur for the non-arrest patient. The frequency and clinical profile of such non-arrest patients has not been well-investigated.

**Methods:**

We undertook a prospective 3-year cohort investigation of patients for whom a smartphone geospatial application was activated for suspected OHCA in four United States communities (total population ~1 million). The current investigation evaluates those patients with an activation for suspected OHCA who did not experience cardiac arrest. The volunteer response cohort included off-duty, volunteer public safety personnel (verified responders) notified regardless of location (public or private) and laypersons notified to public locations. The study linked the smartphone application information with the EMS records to report the frequency, condition type, and EMS treatment for these non-arrest patients.

**Results:**

Of 1779 calls where volunteers were activated, 756 had suffered OHCA, resulting in 1023 non-arrest patients for study evaluation. The most common EMS assessments were syncope (15.9%, *n=*163), altered mental status (15.5%, *n=*159), seizure (14.3%, *n=*146), overdose (13.0%, *n=*133), and choking (10.5%, *n=*107). The assessment distribution was similar for private and public locations. Overall, the most common EMS interventions included placement of an intravenous line (43.1%, *n=*441), 12-Lead ECG(27.9%, *n=*285), naloxone treatment (9.8%, *n=*100), airway or ventilation assistance (8.7%, *n=*89), and oxygen administration (6.6%, *n=*68).

**Conclusions:**

More than half of patients activated for suspected OHCA had conditions other than cardiac arrest. A subset of these conditions may benefit from earlier care that could be provided by both layperson and public safety volunteers if they were appropriately trained and equipped.

**Supplementary Information:**

The online version contains supplementary material available at 10.1186/s12873-023-00849-z.

## Background

Geospatial smartphone application alert systems have been implemented in the past decade to crowdsource community response for out-of-hospital cardiac arrest (OHCA) [1]. The strategy uses an automated notification system keyed to select 9-1-1 dispatch codes to alert nearby volunteers via a smartphone application to respond and deliver care prior to arrival of an organized professional emergency response [1–5]. More recently, the crowdsourcing initiative has expanded from public locations to respond to residential locations in the United States, thereby increasing the potential reach of this strategy with the goal of improving OHCA survival [6].

The primary clinical focus of this strategy has logically been OHCA, where early CPR and defibrillation can be lifesaving. However, dispatch identification of OHCA is imperfect, with approximately half of dispatch-suspected OHCA presenting to EMS with a different primary medical emergency [7]. The frequency and clinical profile of such patients has not been well-investigated. A better understanding of these non-arrest patients who receive a crowdsourcing response could help inform how responders should be trained and/or equipped to provide aid to patients they encounter with conditions other than OHCA.

We undertook an investigation of four United States communities that implemented a Verified Responder program using the PulsePoint smartphone app whereby volunteers were alerted by the app to nearby suspected cardiac arrest in public and residential locations. We hypothesized that the non-arrest patients comprise a spectrum of clinical conditions, some of which may also potentially benefit from this crowdsourcing strategy.

## Methods

### Study population, design and setting

The Verified Responder study is a prospective cohort investigation of volunteer response by off-duty public safety alerted by the PulsePoint app for suspected OHCA [8]. The current investigation focuses on those patients with a PulsePoint alert for suspected OHCA who did not experience cardiac arrest, but rather a different medical condition, based upon subsequent assessment of emergency medical services (EMS). The study occurred in four US communities: Sioux Falls SD, Spokane WA, Spokane Valley WA, and Tualatin Valley OR, from January 1, 2018 through December 31, 2020. Collectively these communities have a population just over 1 million persons (range 105,000 to 547,000) living primarily in urban and suburban areas covering 614 square miles [8]. The incidence of EMS-treated OHCA in these communities range from 45-85 per 100,000 population. The program was approved by each community’s pertinent oversight bodies, and the study was approved by the University of Washington Investigational Review Board. The study was determined to be minimal risk therefore individual consent was not required.

### Verified responder program

The initial Verified Responder Program involving OHCA patients has been reported previously [6]. Briefly, the program involves fire-based first responders practiced in emergency response and lifesaving care to respond while off-duty, often equipped with an AED, to suspected OHCA in public *and* private locations through a geospatial smart phone application integrated with the community 9-1-1 communication center. The current investigation also includes conventional alerts of laypersons for suspected OHCA restricted to public locations.

As part of the Verified Responder Program, each community specified the individual dispatch codes that would trigger a PulsePoint volunteer notification of suspected OHCA. Determination of public vs private location are generated from geolocation services but may be manually adjusted by individual agencies. Locations deemed to be in public locations alerted both laypersons and Verified Responders to respond if the suspected OHCA was within ¼ mile radius. Private location activations increased from ¼ mile radius to a ½ mile radius on October 1, 2018 in an effort to activate more Verified Responders to private residence activations. The notification would not override a phone’s sleep or silence setting initially. All volunteer participants were provided the option to install a silence override when this software upgrade became available in December 2018. In all cases of notification, response was entirely voluntary and at the discretion of the alerted individual. The volunteer response was independent of the conventional on-duty 9-1-1 public safety EMS response in each of these communities.

### Measurement

Information was ascertained using a standard abstraction form from PulsePoint and EMS records. Information abstracted from PulsePoint records included details about the location and time of call, the associated dispatch code, and the number of laypersons and Verified Responders alerted. The information from the EMS prehospital record included patient demographics, the EMS assessment, field care, and transportation status (Additional Table 1). The incident type was categorized based on review of the following EMS record fields: chief complaint, primary impression, secondary impression, and narrative notes. EMS care was abstracted and coded by quality improvement (nonphysician) site specialists through review of the EMS record.

**Table 1:** Initial dispatch codes of PulsePoint activations and top non-OHCA or DOA categories

	SCA - Generic Sudden Cardiac Arrest (*n=*1171)	09E01 - Not breathing at all (*n=*368)	11D01 - Abnormal breathing (PARTIAL obstruction) (*n=*90)	09E02 - Breathing uncertain (AGONAL) (*n=*41)	09D01 - INEFFECTIVE BREATHING (*n=*30)	11E01 - COMPLETE obstruction/ INEFFECTIVE BREATHING (*n=*9)	11D02 - Not alert & 14D01 - Unconscious or Arrest (*n=*6)	12D01 - Not breathing (after Key Questioning) (*n=*6)	Not a PulsePoint CPR push event determinant code. Code changed after initial submission. (*n=*58)
OHCA	24.4% (286)	50.3% (185)	2.2% (2)	17.0% (7)	30.0%(9)	0.0% (0)	16.7% (1)	33.3% (2)	5.2% (3)
DOA	11.2%(132)	21.2%(78)	0.0%(0)	17.0% (7)	0.0%(0)	0.0%(0)	0.0%(0)	0.0%(0)	75.9%(44)
Syncope	12.0%(141)	3.3%(12)	0.0%(0)	9.8%(4)	16.7%(5)	0.0%(0)	16.7% (1)	0.0%(0)	0.0%(0)
Altered Mental Status	13.0%(152)	1.1%(4)	0.0%(0)	4.9%(2)	0.0%(0)	0.0%(0)	0.0%(0)	0.0%(0)	1.7%(1)
Seizure	10.6%(124)	3.0%(11)	2.2%(2)	12.2%(5)	3.3%(1)	0.0%(0)	0.0%(0)	33.3% (2)	1.7%(1)
Overdose	6.7%(78)	12.5%(46)	0.0%(0)	2.4%(1)	20%(6)	0.0%(0)	0.0%(0)	0.0%(0)	3.5%(2)
Choking	0.9%(10)	0.8%(3)	90%(81)	0.0%(0)	0.0%(0)	100%(9)	50.0%(3)	0.0%(0)	1.7%(1)

### Outcomes and analysis

We used descriptive statistics using IBM SPSS v.24 to assess the distribution and compare public versus private location of call types and EMS treatments for non-arrest activations. We used *p* < 0.01 to determine statistical significance given the number of public versus private location comparisons.

## Results

**Fig. 1:**
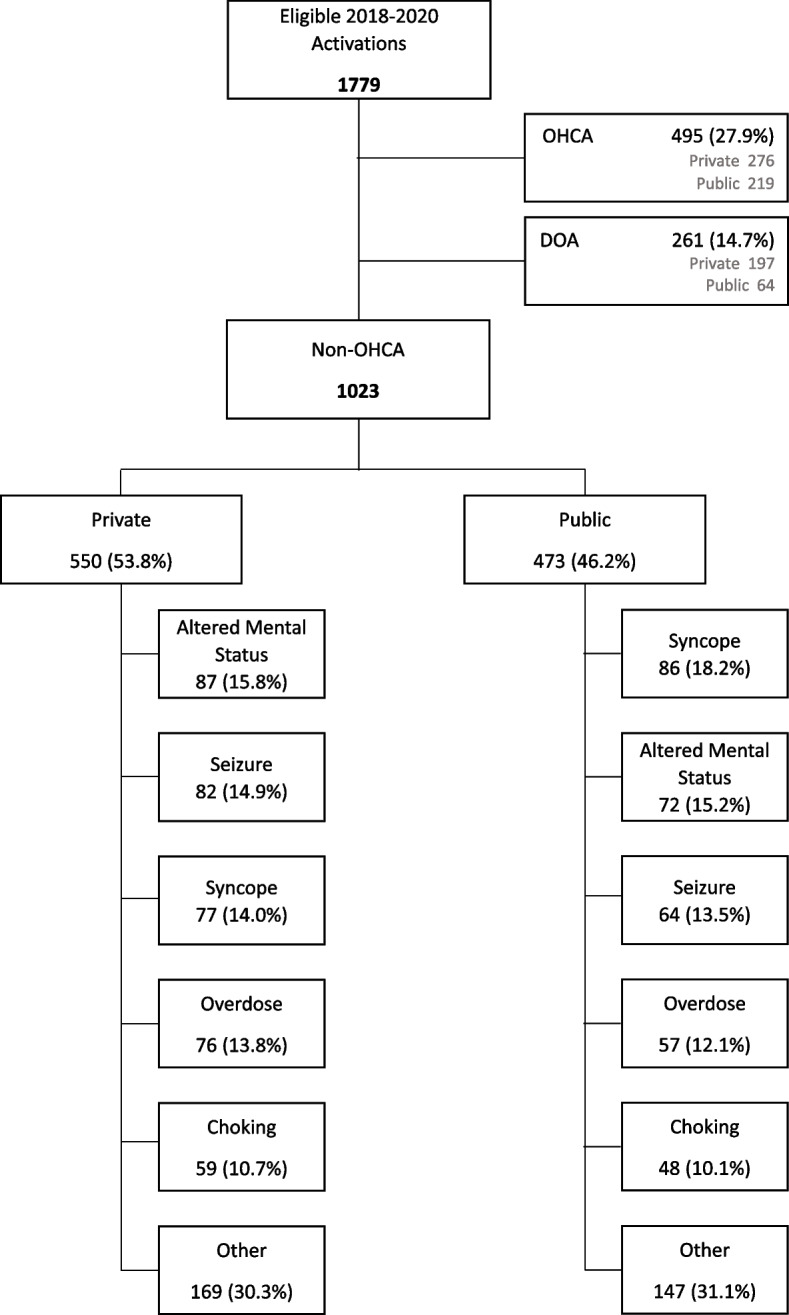
Flow diagram of activations for OHCA and non-OHCA conditions

During the 3-year study period, the application was activated for 1779 9-1-1 calls among the 4 study communities (447 in 2018, 685 in 2019, and 647 in 2020). The 1779 calls produced a total of 4072 notifications (1266 Verified Responder notifications, 2806 layperson notifications). Of these 1779 calls, 495 (27.8%) involved patients who suffered OHCA and received treatment and 261 (14.7%) involved patients who were determined to be DOA and received no attempt at resuscitation, resulting in 1023 patients with an alert (for suspected OHCA) who did not experience OHCA (Figure 1). The dispatch codes were predominantly “sudden cardiac arrest” or different breathing abnormalities for OHCA, DOA, and the 5 most common non-OHCA conditions (Table 1). Of the 1023, the most common condition types based on EMS assessment were syncope (15.9%, *n=*163), altered mental status (15.5%, *n=*159), seizure (14.3%, *n=*146), overdose (13.0%, *n=*133), and choking (10.5%, *n=*107) (Figure 1). The distribution of most patient characteristics and condition types did not significantly differ according to public or private setting location, though we did observe those cases in private location were older, less likely to present with no medical issue (i.e. sleeping), more likely to require an IV, and more likely to require EMS transport (Additional Table 2).

**Table 2:** Characteristics of non-OHCA PulsePoint activations

	Total Activations (*n=*1023)	Private Activations (*n=*550)	Public Activations (*n=*473)	Syncope (*n=*163)	Altered Mental Status (*n=*159)	Seizure (*n=*146)	Overdose (*n=*133)	Choking (*n=*107)	Trauma (*n=*47)
Age years, median (25^th^,75^th^ %)	56(32, 75)	61(34, 79)	52(31, 72)	68(48, 82)	70(49, 84)	45(25, 61)	34(26, 39)	61(23, 77)	58(38, 79)
Female % (n)	43.2(442)	46.0(253)	40.0(189)	46.6(76)	50.9(81)	41.1(60)	33.1(44)	46.7(50)	46.8(22)
Resolved prior to EMS arrival, % (n)	4.1(42)	3.8(21)	4.4(21)	3.1(5)	0.6(1)	2.1(3)	7.5(10)	15.0(16)	2.1(1)
EMS Transport % (n)	76.3(781)	78.9(434)	73.4(347)	75.5(123)	95.6(152)	90.4(132)	85.0(113)	38.3(41)	78.7(37)
*EMS Interventions % (n)*									
IV/IO	43.1(441)	47.1(259)	38.5(182)	39.3(64)	50.3(80)	48.6(71)	60.2(80)	14.0(15)	57.4(27)
ETT	3.3(34)	3.3(18)	3.4(16)	0.6(1)	3.1(5)	1.4(2)	2.3(3)	1.9(2)	21.3(10)
Oxygen	6.6(68)	4.7(26)	8.9(42)	2.5(4)	1.9(3)	2.7(4)	19.5(26)	10.3(11)	8.5(4)
Airway Cleared	5.4(55)	6.0(33)	4.7(22)	0.6(1)	0.6(1)	2.1(3)	6.8(9)	13.1(14)	23.4(11)
12-Lead ECG	27.9(285)	27.3(150)	28.5(135)	56.4(92)	37.1(59)	24.0(35)	12.8(17)	7.5(8)	8.5(4)
Glucose	3.1(32)	4.2(23)	1.9(9)	0.6(1)	3.8(6)	0(0)	0(0)	0(0)	0(0)
Naloxone	9.8(100)	9.6(53)	9.9(47)	0.6(1)	6.3(10)	0(0)	62.4(83)	0(0)	0(0)

	No Medical Issue/ Sleeping (*n=*48)	Other (*n=*40)	Suspected Stroke (*n=*40)	Respiratory (*n=*40)	Alcohol Intoxication (*n=*33)	Diabetic Emergency (*n=*31)	Cardiac Dysrhythmia (*n=*20)	Behavioral/ Mental Health (*n=*12)	COVID-19 (*n=*4)
Age years, median (25^th^,75^th^ %)	59(22, 67)	62(21, 84)	78(65, 87)	69(28, 82)	34(24, 47)	52(40, 66)	65(53, 84)	46(27, 55)	56(32, 65)
Female % (n)	18.8(9)	45.0(18)	47.5(19)	55.0(22)	30.3(10)	41.9(13)	25.0(5)	75.0(9)	100(4)
Resolved prior to EMS arrival, % (n)	2.1(1)	0(0)	2.5(1)	7.5(3)	0(0)	3.2(1)	0(0)	0(0)	0(0)
EMS Transport % (n)	2.1(1)	77.5(31)	100(40)	85.0(34)	66.7(22)	77.4(24)	95.0(19)	66.7(8)	100(4)
*EMS Interventions % (n)*									
IV/IO	0(0)	30.0(12)	72.5(29)	42.5(17)	18.2(6)	87.1(27)	50.0(10)	8.3(1)	50.0(2)
ETT	0(0)	2.5(1)	12.5(5)	12.5(5)	0(0)	0(0)	0(0)	0(0)	0(0)
Oxygen	0(0)	2.5(1)	2.5(1)	30.0(12)	0(0)	3.2(1)	5.0(1)	0(0)	0(0)
Airway Cleared	0(0)	2.5(1)	17.5(7)	15.0(6)	0(0)	6.5(2)	0(0)	0(0)	0(0)
12-Lead ECG	0(0)	17.5(7)	55.0(22)	37.5(15)	9.1(3)	22.6(7)	75.0(15)	8.3(1)	0(0)
Glucose	0(0)	2.5(1)	2.5(1)	0(0)	0(0)	74.2(23)	0(0)	0(0)	0(0)
Naloxone	0(0)	2.5(1)	5.0(2)	2.5(1)	0(0)	3.2(1)	5.0(1)	0(0)	0(0)

Overall, 57% of patients (584/1023) received EMS interventions/diagnostic testing captured by the abstraction, and over three quarters of patients (781/1023) were transferred by EMS to the hospital (Table 2). The most common EMS interventions included placement of an intravenous line (43.1%, *n=*441) and 12-lead ECG acquisition (27.9%, *n=*285). A smaller proportion received naloxone therapy (9.8%, *n=*100), airway or ventilation assistance (8.7%, *n=*89), and glucose administration (3.1%, *n=*32).

## Discussion

In this observational investigation involving four US communities, public safety professionals and laypersons responded to a variety of non-arrest medical conditions as part of a program using a geospatial smartphone application designed to achieve early volunteer notification and action for OHCA. More than half of activations were for medical emergencies other than cardiac arrest in both the public and private settings, highlighting the challenge of precise telephone identification of cardiac arrest. Nonetheless, many of these non-arrest conditions comprised time-sensitive conditions for which early intervention exist that could help limit morbidity or even reduce mortality.

Crowdsourcing volunteers through geospatial smartphone application is a strategy to achieve earlier intervention for persons who suffer cardiac arrest. The use of off-duty public safety professionals as Verified Responders is one approach that has been used to expand the strategy into private locations [6, 8]. Importantly, this group of Verified Responders has advanced training and experience (either certified EMTs or paramedics) that could also be used to treat encountered conditions other than arrest.

We observed a heterogeneous spectrum of non-arrest acute illness that would manifest as unconsciousness and abnormal breathing, criteria typically used by dispatch telecommunicators to identify potential arrest [9]. A prior single-site study from the United States also observed a heterogeneous collection of non-arrest conditions, though there was not a description of subsequent prehospital care [10]. European experiences also indicate that such programs are likely to involve non-arrest patients [1, 4, 5]. The ratio of non-arrest versus arrest cases varies across these systems, likely reflecting differences in triggering dispatch criteria, whether the activation is automated or is gated by human oversight, and the community profile of acute illness. Importantly, these conditions span airway, neurologic, cardiovascular, metabolic, and traumatic conditions, suggesting that the verified responder should be prepared to consider a range of illness types.

With regard to the potential for treatment, the heterogeneous collection of conditions would need to consider a range of therapies. Choking, which accounted for 10.5% of non-arrest cases, is a circumstance where providers trained with basic skills could possibly provide lifesaving care without additional supplies. Several other conditions might also be amenable to early treatment but would require additional equipment, medication, or training. For example, 9.8% were assessed by EMS for suspected drug overdose and received naloxone, suggesting that a “Verified Responder kit” could include a bag valve mask and/or naloxone – treatments that are part of the scope of basic life skills. Other conditions such as seizure, stroke, or hypoglycemia would require additional skills or controlled therapies. Importantly, the need for EMS intervention was variable depending on the underlying condition; for example, choking required EMS intervention in only 13% while drug overdose was treated with naloxone in nearly two-thirds. These proportions help gauge how often the Verified Responders might actually intervene with treatment.

The study has limitations. The investigation provides an assessment of patient illness profile and the treatment opportunity for a strategy designed to achieve early intervention for cardiac arrest. The study did not prospectively attempt to identify non-arrest conditions or implement a treatment kit that would care for these non-arrest patients. However, the information from the current study helps inform the composition of training or treatment that might be useful if a volunteer responder program wanted to provide care for non-arrest patients. Moreover, the distribution of case type can be determined in part at the dispatch level so a system could actively work to use this strategy to involve non-arrest circumstances. Nonetheless, the study did not capture information about potentially-relevant patient acuity such as presenting vital signs or active seizure to gauge illness severity and/or its time sensitivity. Similarly, the study did not capture information about therapies such as hemorrhage control among the 5% of cases with trauma, anticonvulsant administration among the 15% with seizure, or the details of airway clearance which would have enabled more insightful understanding of the implications for the volunteer responder program. Finally, the study did not collect details of response and actions of the verified responders to non-OHCA events, rather just the EMS care. Future investigation should consider a broader and more detailed collection of EMS treatments as well as a survey of the verified responders to understand their perspective about specific treatment opportunities. The investigation occurred in 4 US communities so the profile of patients and subsequent EMS treatments may not be generalizable to all settings. The study did not evaluate some relevant EMS treatments such as the administration of anticonvulsant treatment. The results relied in part of EMS reporting of care. Consequently, some basic treatments such as oxygen administration may not have been comprehensively recorded such that their reported prevalence likely represent a lower boundary estimate.

## Conclusions

More than half of Verified Responder and layperson notifications were for conditions other than cardiac arrest. A subset of these conditions may benefit from earlier care that could be provided by volunteers if they were appropriately trained and equipped. Communities considering this type of crowdsourcing strategy should consider if and how they wish to be involved with the treatment of non-arrest conditions that are commonly encountered during response for suspected cardiac arrest.

## Supplementary Information

**Additional file 1.**

**Additional file 2.**

## Data Availability

The datasets used and/or analyzed during the current study are available from the corresponding author on reasonable request.

## Abbreviations

OHCA: Out-of-hospital cardiac arrest
EMS: Emergency medical services
IV: Intravenous
IO: Intraosseous
ETT: Endotracheal Intubation
AED: Automated external defibrillator
