# Supplementary material for: Characterization of non-cardiac arrest PulsePoint activations in public and private settings
Source: BMC Emerg Med. 2023 Jul 27;23:79. doi: 10.1186/s12873-023-00849-z (PMC10375779; doi:10.1186/s12873-023-00849-z)
Supplement: Supplementary file 1 — Additional file 1. [file 12873_2023_849_MOESM1_ESM.docx]

| **Abstraction Variables** |
| --- |
| PulsePointMasterIncident# |
| AgencyIncidentNumber |
| AgencyEventNumber |
| Address |
| Call Received Date/Time |
| Dispatch Code |
| Chief Complaint |
| Primary Impression |
| Sex |
| Age |
| Intervention: IV/IO |
| Intervention: Intubation |
| Intervention: O2 |
| Intervention: Airway cleared |
| Intervention: 12-Lead |
| Intervention: ASA |
| Intervention: Nitro |
| Intervention: Epi |
| Intervention: Glucose |
| Intervention: Narcan |
| Intervention: Stroke Alert |
| Transported? Yes/No |
| If known, did issue resolve prior to VR arrival? |
| Narrative Notes |
