# Supplementary material for: Characterization of non-cardiac arrest PulsePoint activations in public and private settings
Source: BMC Emerg Med. 2023 Jul 27;23:79. doi: 10.1186/s12873-023-00849-z (PMC10375779; doi:10.1186/s12873-023-00849-z)
Supplement: Supplementary file 2 — Additional file 2. [file 12873_2023_849_MOESM2_ESM.docx]

|  | Total Activations (n=1023) | Private Activations (n=550) | Public Activations (n=473) |
| --- | --- | --- | --- |
| Age years, median (IQR) | 56(32, 75) | 61(34, 79) | 52(31, 72)* |
| Female % (n) | 43.2(442) | 46.0(253) | 40.0(189) |
| Resolved prior to EMS arrival, % (n) | 4.1(42) | 3.8(21) | 4.4(21) |
| EMS Transport % (n) | 76.3(781) | 78.9(434) | 73.4(347)* |
| *EMS Interventions % (n)* |  |  |  |
| IV/IO | 43.1(441) | 47.1(259) | 38.5(182)* |
| ETT | 3.3(34) | 3.3(18) | 3.4(16) |
| Oxygen | 6.6(68) | 4.7(26) | 8.9(42) |
| Airway Cleared | 5.4(55) | 6.0(33) | 4.7(22) |
| 12-Lead ECG | 27.9(285) | 27.3(150) | 28.5(135) |
| Glucose | 3.1(32) | 4.2(23) | 1.9(9) |
| Naloxone | 9.8(100) | 9.6(53) | 9.9(47) |
| *Category* |  |  |  |
| Syncope | 15.9(163) | 14.0(77) | 18.2(86) |
| Altered Mental Status | 15.5(159) | 15.8(87) | 15.2(72) |
| Seizure | 14.3(146) | 14.9(82) | 13.5(64) |
| Overdose | 13.0(133) | 13.8(76) | 12.1(57) |
| Choking | 10.5(107) | 10.7(59) | 10.1(48) |
| Trauma | 4.6(47) | 5.1(28) | 4.0(19) |
| No Medical Issue/ Sleeping | 4.7(48) | 3.1(17) | 6.6(31)* |
| Other | 3.9(40) | 3.6(20) | 4.2(20) |
| Suspected Stroke | 3.9(40) | 4.7(26) | 3.0(14) |
| Respiratory | 3.9(40) | 3.1(17) | 4.9(23) |
| Alcohol Intoxication | 3.2(33) | 2.7(15) | 3.8(18) |
| Diabetic Emergency | 3.0(31) | 4.0(22) | 1.9(9) |
| Cardiac Dysrhythmia | 2.0(20) | 2.2(12) | 1.7(8) |
| Behavioral/ Mental Health | 1.2(12) | 1.6(9) | 0.6(3) |
| COVID-19 | 0.4(4) | 0.5(3) | 0.2(1) |

*Indicates p-value <0.01
